# Behavioral benefits of GSK-3β inhibition and state-dependent microtubule signatures in the *Fmr1*-KO mouse

**DOI:** 10.3389/fnins.2025.1643439

**Published:** 2025-10-02

**Authors:** John Kealy, Charlotte Callaghan, Aimée Freeburn, Aoife Thornton, Chris Greene, Beatrice Garrone, Claudio Milanese, Massimiliano Bianchi

**Affiliations:** ^1^Ulysses Neuroscience Ltd., Dublin, Ireland; ^2^Trinity College Institute of Neuroscience, Lloyd Institute, Trinity College Dublin, Dublin, Ireland; ^3^Smurfit Institute of Genetics, Trinity College Dublin, Dublin, Ireland; ^4^Angelini Pharma S.p.A., Rome, Italy

**Keywords:** fragile X syndrome, FMR1, microtubule dynamics, alpha-tubulin, GSK-3β, X-linked disorder

## Abstract

Glycogen-synthase-kinase-3β (GSK-3β) and microtubule dynamics are implicated in Fragile X syndrome (FXS). We examined behaviors and hippocampal *α*-tubulin post-translational modifications (PTMs) in *Fmr1*-KO male mice without and with chronic administration of the GSK-3β inhibitors SB216763 (30 mg/kg, i.p.) and AF3581 (10 mg/kg, i.p.). *Fmr1*-KO male mice and wild-type (WT) were evaluated in the open field, marble-burying, elevated-plus-maze (EPM), novel-object-recognition (NOR) and three-chamber sociability test (3-CST); acetylated *α*-tubulin (Acet/Total-Tub) and tyrosinated/detyrosinated *α*-tubulin (Tyr/Glu-Tub) ratios were then analyzed. *Fmr1*-KO male showed hyperactivity, excessive marble burying and impaired NOR; Acet/Total-Tub was elevated and Tyr/Glu-Tub reduced vs. WT, indicating reduced microtubule dynamics. In a mixed-sex cohort bred female WT displayed lower Acet/Total-Tub and increased Tyr/Glu-Tub vs. male WT. The *Fmr1*-KO-associated decrease in Tyr/Glu-Tub was consistent across sexes. FMRP and synaptic markers were also analyzed in this cohort, spinophilin was found increased in both male and female *Fmr*1-KO. *Fmr1*-heterozygous females showed no molecular alterations, supporting the protective role of FMRP. *Fmr1*-KO male mice received vehicle or GSK-3β inhibitors and were tested in behavioral assays followed by *α*-tubulin PTMs analysis. Daily vehicle injections appeared to abolish baseline differences in hyperactivity, marble burying and *α*-tubulin PTMs. Under these conditions both inhibitors reduced marble burying. SB216763 normalized social discrimination in 3-CST, while AF3581 only produced a non-significant positive trend. Neither compound altered *α*-tubulin PTMs. These results show that GSK-3β inhibition has anti-perseverative and pro-social effects in *Fmr1*-KO male mice. However, behavioral and molecular endpoints, such as *α*-tubulin PTMs, appear to be sensitive to both genetic background and experimental procedures.

## 1 Introduction

Fragile X syndrome (FXS), an X-linked neurodevelopmental disorder, is the most common inherited cause of intellectual disability and autism spectrum disorder associated with symptoms including intellectual disability, developmental delay, autism-like behaviors, anxiety, and seizures in about 20% of patients (Hagerman et al., 2017). FXS is caused by a massive expansion of a CGG motif in the Fragile X Messenger Ribonucleoprotein 1 (*FMR1*) gene (locus Xq27.3), increasing from a normal range of <50 repetitions of the CGG triplet to 200 + repetitions (Darnell and Klann, 2013). This causes hypermethylation of CpG sites in the FMR1 promoter region, which leads to transcriptional silencing of the gene and consequent loss of Fragile X Messenger Ribonucleoprotein (FMRP) protein expression. This lack of FMRP prevents ribosomal translocation of numerous mRNA targets which are normally downregulated by FMRP leading to increased translation of a range of downstream genes (Darnell and Klann, 2013; Darnell et al., 2011). In the brain, FMRP is crucial in normal neurodevelopment and synaptic plasticity throughout the lifespan (Darnell and Klann, 2013). Individuals with FXS not only can have extensive cognitive and behavioral symptoms but also show structural changes; magnetic resonance imaging (MRI) shows that FXS is associated with reductions in gray matter across several brain regions (Sandoval et al., 2018). Structural changes begin to manifest in the FXS brain by 6 months of age and changes in caudate nucleus volume positively correlate with repetitive behaviors later in childhood (Shen et al., 2022). As females have a second copy of the *FMR1* gene, they usually present with milder symptoms compared to males with FXS and this is reflected in the neuroanatomy with females with FXS having larger gray matter volumes compared to males (Lee et al., 2022).

Besides its canonical role in translational control, FMRP interacts, directly or indirectly, with several microtubule-associated proteins and regulates local translation of *β*-actin and *α*-tubulin transcripts (Lu et al., 2004). Microtubule “dynamic instability” is essential for growth-cone steering, axonal transport and spine pruning; dampening this dynamism is increasingly linked to neurological disorders characterized by synaptic pathology (Barbiero et al., 2022). Thus, microtubules are strongly implicated in a range of neurodevelopmental disorders as they are key players during neuronal development in the embryo (Lasser et al., 2018) and targeting them as a therapeutic strategy appears to be a viable strategy for treating neurodevelopmental disorders (Lasser et al., 2018; Bonini et al., 2017). In *Fmr1* KO mice, microtubules are resistant to nocodazole-induced shifts toward a more dynamic state (Lu et al., 2004). This opens up the possibility of using other strategies to target microtubules in FXS in order to promote a more dynamic microtubule state and thus increase levels of synaptic plasticity. Two *α*-tubulin post-translational modifications (PTMs) serve as operational read-outs of microtubule dynamics: (i) detyrosination, quantified as the tyrosinated/detyrosinated ratio (Tyr/Glu-Tub), and (ii) acetylation, quantified as the acetylated/total ratio (Acet/Total-Tub). Reduced Tyr/Glu-Tub together with elevated Acet/Total-Tub marks less dynamic, more stabilized microtubules. We and others have reported this *α*-tubulin PTM signature in brain regions of experimental models and clinical samples of patients affected by neurological disorders characterized by synaptic pathology (Lu et al., 2004; Bianchi and Baulieu, 2012).

The understanding of FXS in humans has been greatly aided by the *Fmr1*-knock-out (KO) mouse model. The *Fmr1-*KO mouse has been extensively characterized with variable phenotypes described in terms of behavior and electrophysiology. Behaviorally, these mice show increases in locomotor activity, changes in sociability, cognitive impairments, and the presence of audiogenic seizures (Kazdoba et al., 2014). From a neurophysiological perspective, *Fmr1*-KO mice show distinct impairments in synaptic plasticity including enhancement of metabotropic glutamate receptor-mediated (mGluR-mediated) long-term depression (LTD) (Huber et al., 2002; Niere et al., 2012), along with morphological changes in dendritic spines – longer and thinner spines instead of mature mushroom-shaped spines associated with mature synapses (Comery et al., 1997; Nimchinsky et al., 2001). These changes in dendritic spines are also observed in *post-mortem* analysis of human FXS brain tissue (Rudelli et al., 1985; Wisniewski et al., 1991; Irwin et al., 2000). However, phenotype penetrance in *Fmr1*-KO mice is notoriously sensitive to genetic background and procedural stress. Baker et al. showed that robust spatial-learning deficits emerge on an albino C57BL/6 J background but not on others (Baker et al., 2010), while Spencer et al. demonstrated that hyperactivity, marble burying and sociability vary dramatically across six common inbred strains (Spencer et al., 2011).

FMRP loss elevates glycogen-synthase-kinase-3 (GSK-3) activity. In *Fmr1*-KO brain the activity-limiting Ser-phosphorylation of GSK-3 is reduced (Mines and Jope, 2011; Min et al., 2009). Pharmacological inhibition of GSK-3 with lithium, which promotes Ser-phosphorylation of the kinase, protects *Fmr1*-KO mice against audiogenic seizures and attenuates hyperlocomotion (Min et al., 2009; Yuskaitis et al., 2010); dietary lithium also modestly improves 3-CST sociability (Liu et al., 2011). A 2008 open-label study suggested lithium is safe and potentially beneficial in people with FXS (Berry-Kravis et al., 2008), but newer, more selective GSK-3 inhibitors have since been developed and may offer a better therapeutic window (Arciniegas Ruiz and Eldar-Finkelman, 2021). Selective inhibition of GSK-3β, specifically using SB216763, have also had success in treating the behavioral and physiological phenotypes observed in *Fmr1*-KO mice. Treatment with SB216763 at 30 mg/kg (i.p.) was protective against audiogenic seizures and treatment at 4 mg/kg (i.p.) reduced hyperlocomotion in *Fmr1* KO mice (Min et al., 2009). More recently, acute treatment with SB216763 (30 mg/kg; i.p.) resulted in a reduction in wild running, suppression of audiogenic seizures, and a reversal of abnormal spine morphology (Westmark et al., 2021). There is evidence that chronic treatment with SB216763 can have long-lasting effects on learning and memory in *Fmr1*-KO mice; 2 mg/kg administered every second day across a 14-day period significantly improved performance in two hippocampal-dependent tasks tested 2 weeks later (Guo et al., 2012). This treatment regime also improved phosphorylated GSK-3β levels and restored hippocampal neurogenesis in *Fmr1* KO mice (Guo et al., 2012). AF3581, a potent ATP-competitive inhibitor bearing an N-[(1-alkyl-piperidin-4-yl)methyl]-1H-indazole-3-carboxamide core, was recently described as an advancement over existing GSK-3β inhibitors owing to its nanomolar potency, high plasma stability and overall drug-like properties (Capurro et al., 2020). AF3581 also recently showed efficacy in correcting the behavioral phenotype observed in *Fmr1* KO mice, with improvements in locomotor activity, sensorimotor gating, and sociability all found after chronic treatment (Porceddu et al., 2021). Its efficacy in the *Fmr1*-KO model had not been compared directly with SB216763. On a molecular level, GSK-3β is a well-known upstream controller of microtubule dynamics (Barnat et al., 2016). Thus, neurite growth in neurons requires inactivation of GSK-3β to promote microtubule organization (Zhou et al., 2004). Against the backdrop of variability in phenotyping Fmr1-KO mice and the need to explore new treatments for FXS, we pursued three main aims using distinct cohorts of animals: (i) Baseline characterization - Establish a comprehensive behavioral profile and molecular characterization of *α*-tubulin PTMs in adult male Fmr1-KO mice; (ii) Sex-dependent molecular analysis – Investigate genetic and sex-related differences in *α*-tubulin PTMs, FMRP, and synaptic markers (spinophilin, synaptophysin, PSD-95) using a mixed-sex cohort generated from Fmr1-KO male × Fmr1-heterozygous female breeding; (iii) Pharmacological intervention – Assess whether a 10-day systemic regimen of SB216763 or AF3581 can improve abnormal behaviors and modulate *α*-tubulin PTMs in male Fmr1-KO mice.

## 2 Materials and methods

### 2.1 Mouse model

A B6.129P2-*Fmr1^tm1Cgr^*/J mouse colony was established at Trinity College Dublin using animals from The Jackson Laboratory [JAX stock #003025; (The Dutch-Belgian Fragile X Consortium, 1994)]. Age- and sex-matched wild-type (WT) littermates from the same litters were used as controls in all behavioral and biochemical experiments. Two breeding schemes were employed: (i) Baseline/treatment cohorts: heterozygous females (X^KO X^WT) were obtained by back-crossing *Fmr1*-KO males with C57BL/6 J WT females; mating these heterozygous females with WT males produced WT and Fmr1-KO male littermates for the drug-naïve and GSK-3β inhibitor studies; (ii) Mixed-sex cohort for molecular studies: heterozygous carrier females (X^KO X^WT), generated in a first back-cross (*Fmr1*-KO male × C57BL/6 J female), were mated with *Fmr1*-KO males (X^KO Y). This cross produced all four genotypes in the same litter: homozygous KO females (X^KO X^KO), heterozygous females (X^KO X^WT), KO males (X^KO Y) and WT males (X^WT Y). Mice were housed under a 12 h light/dark cycle with food and water ad libitum, in full compliance with European Directive 2010/63/EU and Irish Statutory Instrument No. 543 of 2012. All procedures were approved by the Trinity College Dublin Animal Research Ethics Committee and authorized by the Health Products Regulatory Authority, with veterinary supervision throughout.

### 2.2 Experimental design

**Figure 1:**
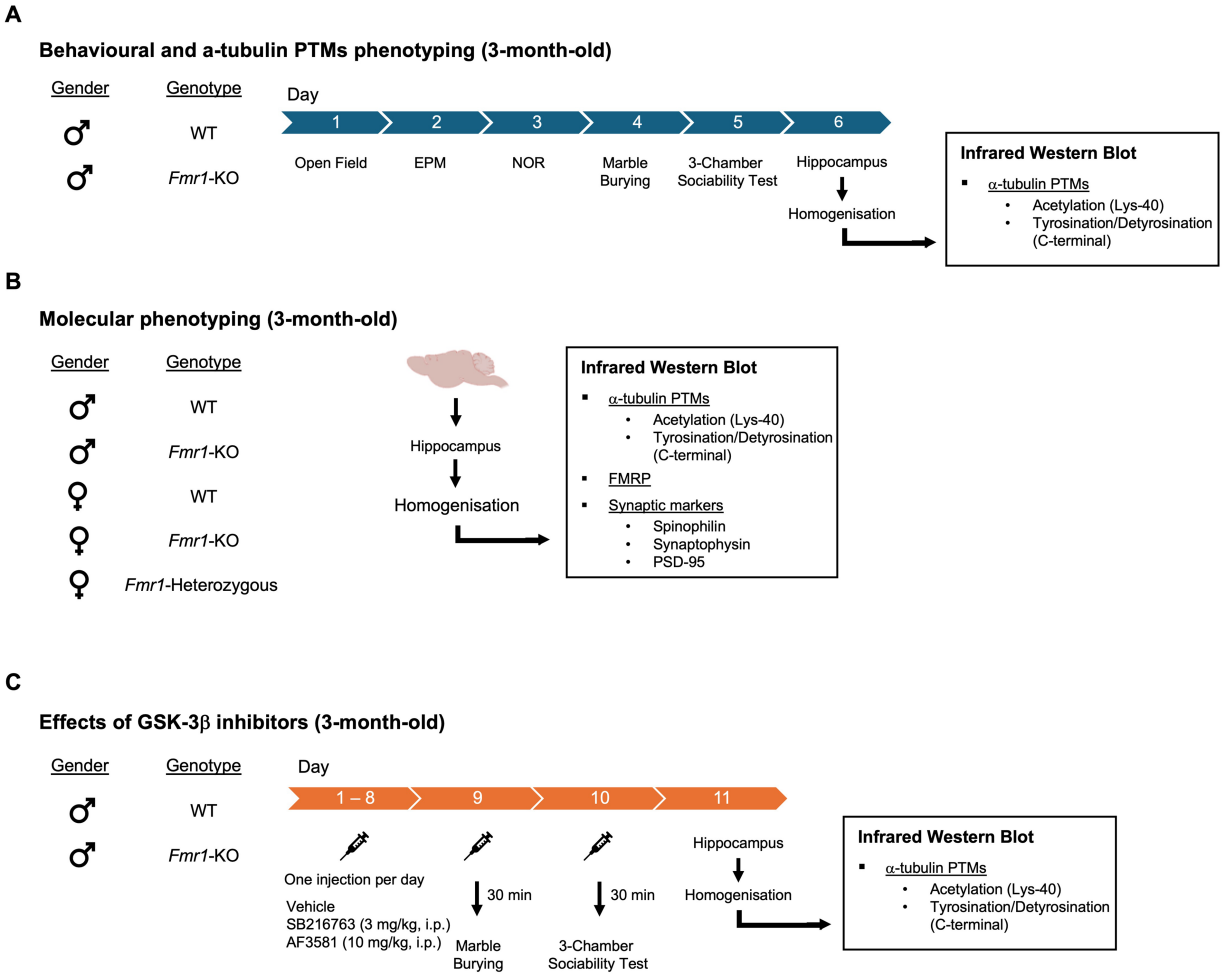
Experimental design (see also main text). Three experimental cohorts were generated for this study. **(A)** Behavioral and *α*-tubulin PTMs phenotyping - A cohort of 3-month-old male mice, WT and *Fmr1*-KO, was used to assess baseline behavioral and *α*-tubulin PTMs phenotypes. Behavioral assays included the open field, elevated plus maze, novel object recognition, marble burying, and 3-chamber sociability test. Mice were sacrificed 24 h after the last behavioral test for molecular analysis of hippocampal tissue. **(B)** Molecular phenotyping - A separate mixed-sex cohort was used to investigate sex-dependent molecular changes in hippocampal lysates. The cohort included five genotypes: WT males, *Fmr1*-KO males, WT females, *Fmr1*-heterozygous females, and *Fmr1*-KO females. Molecular analysis included: *α*-tubulin PTMs, FMRP and synaptic markers. **(C)** Effects of GSK-3β inhibitors – A third of 3-month-old male mice, WT and *Fmr1*-KO, was used to evaluate the effects of GSK-3β inhibition on behavioral and molecular endpoints. WT and *Fmr1*-KO mice received daily intraperitoneal (i.p.) injections of either SB216763 (3 mg/kg) or AF3581 (10 mg/kg) for 10 consecutive days. On Day 9, mice were tested in the marble burying task, and on Day 10 in the 3-chamber sociability test, performed 30 min post-treatment. Mice were sacrificed 24 h after the final injection for molecular analysis of hippocampal tissue.

Three experimental cohorts were generated for this study. A schematic overview of the designs, including mouse genotypes, ages, treatments, and behavioral or molecular endpoints, is provided in Figure 1. A cohort of 3-month-old male mice (WT and *Fmr1*-KO) was used to assess baseline behavioral and hippocampal *α*-tubulin phenotypes. Mice underwent a battery of behavioral tests administered on consecutive days, with no more than one test per day. The test battery included the open field, elevated plus maze, novel object recognition, marble burying, and 3-chamber sociability test. Mice were sacrificed 24 h after the last behavioral test for molecular analysis of hippocampal tissue (Figure 1A). A separate mixed-sex cohort was used to investigate sex-dependent molecular changes in hippocampal lysates. Mice were sacrificed at 3 months of age, and the cohort included five genotypes: WT males, *Fmr1*-KO males, WT females, *Fmr1*-heterozygous females, and *Fmr1*-KO females (Figure 1B). A third cohort of 3-month-old male mice (WT and *Fmr1*-KO) was used to evaluate the effects of GSK-3β inhibition on behavioral and molecular endpoints. WT and *Fmr1*-KO mice received daily intraperitoneal (i.p.) injections of either vehicle, SB216763 (3 mg/kg) or AF3581 (10 mg/kg) for 10 consecutive days. On Day 9, mice were tested in the marble burying task, and on Day 10 in the 3-chamber sociability test, performed 30 min post-treatment. Mice were sacrificed 24 h after the final injection for molecular analysis of hippocampal tissue (Figure 1C).

### 2.3 Drug treatments

SB216763 and AF3581 were provided by Angelini Pharma. The compunds were prepared daily for i.p. injections in the appropriate vehicle (10% PEG400 and 10% Tween 80 in saline). All mice were divided into three treatment groups: (1) SB216763 (30 mg/kg; i.p.); (2) AF3581 (10 mg/kg; i.p.); and (3) vehicle (i.p.). All mice were treated once daily for 10 days with their respective treatment. There were 10 mice in each treatment group and all treatments were delivered at the start of the light phase each morning in a dedicated procedure room. Body weights were recorded every day for dosage calculations and welfare monitoring. The doses of SB216763 and AF3581 were selected based on previous literature (Min et al., 2009; Westmark et al., 2021; Guo et al., 2012; Capurro et al., 2020; Porceddu et al., 2021) as well as internal studies conducted by Ulysses Neuroscience Ltd. and Angelini Pharma. Notably, AF3581 has been shown to increase phosphorylation of hippocampal GSK-3β (Ser9) at the dose of 10 mg/kg (p.o.) without affecting total GSK-3β expression (Porceddu et al., 2021).

### 2.4 Behavioral measures

Prior to any behavioral testing, all mice underwent daily handling by the experimenter to reduce stress and maximize performance in the various tasks. For drug-naïve animals, they underwent a battery of behavioral tests on subsequent days with no more than one test per day. For drug-treated animals, behavioral testing was performed on Days 9 and 10 of chronic treatment (Marble Burying Test and 3-Chamber Sociability Test respectively), with all testing occurring 30 min post-treatment for each mouse. Mice were transferred from the procedure room in a clean transport cage to the behavior room and were returned to their home cage immediately after testing. All apparatus were cleaned with 70% ethanol between trials and between individual mice, allowing ethanol to completely evaporate before introducing an animal into the apparatus.

#### 2.4.1 Open field test

Locomotor activity and exploratory behavior were assessed in an open field arena (58 cm × 33 cm × 19 cm), which was divided into 10 cm × 10 cm squares using a grid drawn beneath the floor of the arena. Each mouse was placed in the center of the arena and allowed to explore freely for 3 min. The number of squares crossed—defined as all four paws entering a new square—was manually counted by an experimenter blind to genotype, as previously described (Murray et al., 2013). To assess anxiety-like behavior, the arena was subdivided into an inner zone and a peripheral zone. The total time each mouse spent in the inner zone was recorded using a stopwatch. No video recording system was used for this test. The arena was cleaned with 70% ethanol between animals to eliminate olfactory cues.

#### 2.4.2 Elevated plus maze

Mice were allowed to explore an elevated plus maze (Ugo Basile, Italy) for 8 min using a protocol adapted from (Walf and Frye, 2007). Briefly, the apparatus consists of two open arms (30 cm × 5 cm) and two closed arms (30 cm × 5 cm) with 15 cm-high walls, elevated 60 cm above the floor. A camera placed directly above the maze recorded each session. Mice were placed at the center of the maze facing an open arm at the beginning of each trial. Video recordings were manually scored to extract the time spent in each of the four arms. The open arm index was calculated as the time spent in the open arms divided by the total time spent in both open and closed arms: Open Arm Index = Time in Open Arms/(Time in Open Arms + Time in Closed Arms). This index was used to compare anxiety-like behavior between the two strains.

#### 2.4.3 Novel object recognition test

The open field apparatus described in section 2.3.1 was used to present pairs of objects to the mice. In the sample phase, mice were exposed to a pair of identical objects (plastic culture flasks filled with sodium chloride) and allowed to explore them for 5 min as previously described (Greene et al., 2018). Briefly, mice were then returned to their home cage for a 1-h retention interval. In the test phase, each mouse was reintroduced into the arena for 5 min with one familiar object from the sample phase and one novel object (a plastic tube filled with colored liquid). A camera was positioned above the arena and all sessions were recorded. Videos were manually scored by an experimenter blind to genotype. The number of nose touches directed at each object was counted for each trial. The arena and all objects were cleaned with 70% ethanol between mice and between trials. The discrimination index was calculated as the difference in exploration time between the novel and familiar object, divided by the total exploration time for both objects: Discrimination Index = (Time with Novel Object – Time with Familiar Object)/(Time with Novel Object + Time with Familiar Object).

#### 2.4.4 Marble burying test

The marble burying test was performed as previously described (Deacon, 2006). Briefly, a large cage (46 cm × 35 cm × 20 cm) was filled with 5 cm of wood chip bedding (Lignocel, Germany), which was gently leveled prior to each session. A grid of 20 evenly spaced glass marbles (1.5 cm in diameter) was arranged on the surface of the bedding in 4 rows of 5 marbles. Each mouse was individually placed in the center of the apparatus and allowed to explore freely for 30 min. At the end of the session, mice were returned to their home cage. The number of marbles buried—defined as having at least two-thirds of their surface covered by bedding—was manually scored by an experimenter blind to genotype. A camera was not used in this assay. The bedding was stirred and replaced between animals to ensure consistent starting conditions.

#### 2.4.5 3-chamber sociability test

The 3-chamber sociability apparatus (Ugo Basile, Italy) consisted of three interconnected Plexiglas chambers, each measuring 20 cm × 40 cm × 22 cm (length × width × height). The side chambers contained wire mesh holding cages to enclose stimulus mice. In the Habituation trial, the test mouse was allowed to freely explore all three chambers for 5 min. In Trial 1 (Sociability), a stranger mouse (male, age- and strain-matched) was placed in one holding cage, while the other cage remained empty. The test mouse explored the apparatus for 10 min. In Trial 2 (Social Novelty), a second novel stranger was placed in the previously empty cage, and the test mouse again explored for 10 min. A camera mounted above the apparatus recorded all trials, and behavior was automatically tracked using ANY-Maze software. ANY-Maze was used to measure: (i) total distance moved across all trials; and (ii) time spent in each chamber during Trials 1 and 2. The Discrimination Index (DI) was calculated for each trial to quantify preference: DI = (Time in Stranger-Chamber – Time in Empty/Familiar Chamber)/(Time in Stranger-Chamber + Time in Empty/Familiar Chamber). For Trial 1, the “empty chamber” was used; for Trial 2, the “familiar stranger chamber” was used.

### 2.5 Infrared Western blot analysis

Animals were sacrificed by decapitation, and the hippocampus was dissected out. This was immediately frozen on crushed dry ice and stored at −80°C. Hippocampal tissue was homogenized in Lysis Buffer containing protease inhibitor cocktail. Hippocampal samples were weighed individually using an analytical balance (Max Cap: 410 × g Readability: 0.001 × g). For every 1 mg of tissue, 6.67 μL of lysis buffer (Tris–HCl 5 mM + EGTA 2 mM) containing a 1:50 dilution of proteinase inhibitor cocktail (PIC; Sigma - P8340) added to the sample. Tissue was homogenized using a Q125 sonicator (QSONICA LLC - QSONQ55UK-220) at 8–9 amplitude microns in 15-s intervals for no more than a total of 30 s. Performance of a Bradford assay (Fisher Scientific - 10495315) enabled equalization of sample protein concentrations as described previously (Bianchi and Baulieu, 2012).

#### 2.5.1 Analysis of α-tubulin PTMs

*α*-tubulin PTMs were measured as described previously (Bianchi and Baulieu, 2012). Briefly, samples were loaded at 0.15 μg of total protein concentration to NuPAGE™ 10%, Bis-Tris, 1.0 mm, Midi Protein Gel, 26-well (Invitrogen, 10,115,312) followed by transfer to PVDF membrane (Sigma - IPFL00010). Acetylated *α*-tubulin (Acet-Tub) and Total *α*-tubulin (TOT-Tub) were detected on the same blot. Acetylated *α*-tubulin (Acet-Tub) was detected by incubating the membranes with Anti-Tubulin, Acetylated antibody produced in mouse [clone: 6-11B-1] (Sigma - T6793) at a concentration of 1:6,000 overnight at 4°C. The membranes were then washed three times for 10 min each with PBS-T 0.1%; and then incubated with secondary antibody donkey anti-mouse IgG 680RD (Li-Cor; 926–68,072) at 1:10,000 for 1 h at RT. Total *α*-tubulin (TOT-Tub) was detected using anti-TUBA Rabbit Monoclonal Antibody [clone: EP1332Y] (Abcam; ab52866) at 1:2, overnight at 4°C. The membranes were then washed three times for 10 min each with PBS-T 0.1% and then incubated with secondary antibody donkey anti-rabbit IgG 800CW (Li-Cor; 926–32,213) at 1:10,000 for 1 h at RT.

Tyrosinated *α*-tubulin (Tyr-Tub) and detyrosinated *α*-tubulin (Glu-Tub) were detected on the same blot. Tyr-Tub was detected by incubating the membranes with the monoclonal anti-tubulin, tyrosine antibody [clone: TUB-1A2] (Sigma - T9028) at 1:500 overnight at 4°C. The membranes were then washed three times for 10 min each with PBS-T 0.1%; and then incubated with and then incubated with secondary antibody donkey anti-mouse IgG 680RD (Li-Cor; 926–68,072) at 1:10,000 for 1 h at RT. Glu-Tub was detected using anti *α*-tubulin rabbit monoclonal antibody [clone; RM444], detyrosinated (Fisher Scientific; 17,846,771) at 1:500 overnight at 4°C. The membranes were then washed three times for 10 min each with PBS-T 0.1%; and then incubated with and then incubated with secondary antibody donkey anti-rabbit IgG 800CW (Li-Cor; 926–32,213) at 1:10,000 for 1 h at RT. Acet-Tub was expressed as a ratio of TOT-Tub, and Tyr-Tub was expressed as a ratio of Glu-Tub. These ratios are reflective of microtubule dynamics, with a high Acet/Total *α*-tubulin ratio and low Tyr/Glu ratio being associated with reduced microtubule dynamics. All data were normalized to the average ratios of the control group and expressed as % of control for statistical analysis.

#### 2.5.2 Analysis of FMRP

Mouse hippocampal samples were loaded at 7.5 μg of total protein concentration to NuPAGE™ 10%, Bis-Tris, 1.0 mm, Midi Protein Gel, 26-well (Invitrogen, 10,115,312) followed by transfer to polyvinylidene fluoride (PVDF) membrane (Sigma; IPFL00010). Following blocking, the membranes were incubated overnight at 4°C with primary antibodies: FMRP (Cell Signaling, U.S.A. 4317S, 1:1,000) and GAPDH [6C5] (AbCam; Ab8245, 1:5,000). Membranes were washed three times for 10 min each with PBS-T (0.1% Tween-20 in PBS) incubated in secondary antibodies for 1 h at Room Temperature (RT): Goat anti-Rabbit IgG 680RD (Li-Cor; 926–6,807, 1:2,000) and Goat anti-Mouse IgG 800CW (Li-Cor; 926–32,210, 1:5,000). Membranes were washed again three times for 10 min each with PBS-T 0.1% before being scanned on the CLx Odyssey imaging system (Li-Cor, U.S.A.). All data were normalized to the average of the control group for statistical analysis.

#### 2.5.3 Analysis of synaptic markers

All synaptic marker were analyzed on the same blot and normalized separately to a loading control (GAPDH) through the use of two fluorescent channels and utilizing separation of molecular weights of each target. Samples were loaded at 25 μg of total protein concentration to NuPAGE™ 10%, Bis-Tris, 1.0 mm, Midi Protein Gel, 26-well (Invitrogen, 10,115,312) followed by transfer to PVDF membrane (Sigma - IPFL00010). Following blocking, the membranes were incubated overnight at 4°C with primary antibodies: PSD-95 [6G6-1C9] (Invitrogen; Ma1-045, 1:2,000), Spinophilin (Sigma; AB5669, 1:750), Synaptophysin (AbCam; ab32127, 1:1,500), and GAPDH [6C5] (AbCam; Ab8245, 1:5,000). The membranes were then washed three times for 10 min each with PBS-T 0.1%; and then incubated with secondary antibodies: Goat anti-Mouse IgG 680RD (Li-Cor; 926–68,070, 1:5,000), and Goat anti-Rabbit IgG 800CW (Licor; 926–32,211, 1:2,500) for 1 h at RT. PSD-95, Spinophilin, and Synaptophysin were normalized to the expression of GAPDH on the same blots.

### 2.6 Data processing and statistical analysis

Statistical comparisons were made between groups using unpaired *t*-tests for comparing two groups, and ANOVAs to compare more than two groups. Where multiple timepoints from the same animals were being compared, a repeated-measures ANOVA was used. ANOVAs were followed by Fisher’s LSD pairwise comparisons with *p* < 0.05 set as the level for significance. All statistical tests were performed using InVivoStat (Version 4.2.0) and all data were graphed using Prism 5 for Mac OSX (Version 5.0b, GraphPad Software Inc., USA).

## 3 Results

### 3.1 Behavioral characterization of *Fmr1*-KO mice

**Figure 2:**
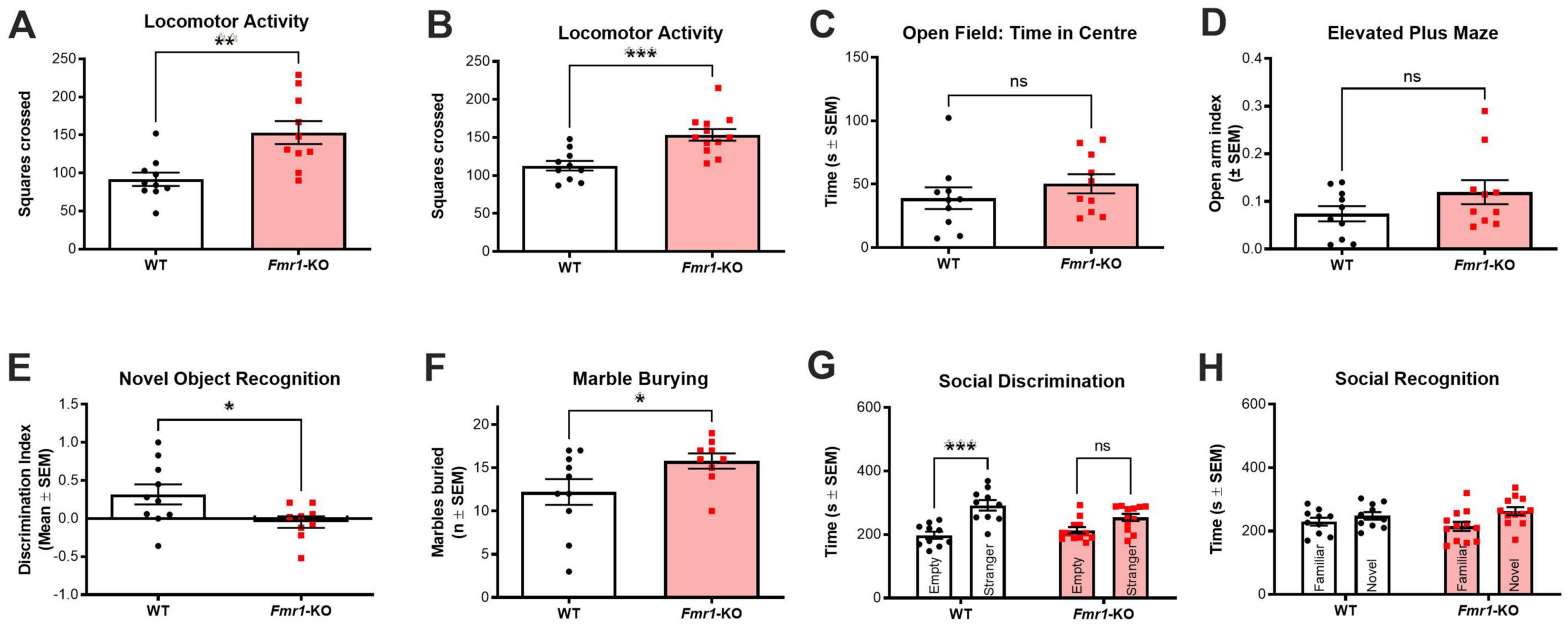
Behavioral characterization of drug-free *Fmr1*-KO mice. **(A,B)** *Fmr1*-KO mice (*n* = 10–12) show significant and consistent hyperlocomotion compared to WT controls (*n* = 10) in the open field across two different cohorts of animals. **(C)** *Fmr1*-KO mice (*n* = 10) and WT controls (*n* = 10) spent similar amounts of time in the center zone of the open field. **(D)** *Fmr1*-KO mice (*n* = 10) and WT controls (*n* = 10) spent similar amounts of time on the open arms of the elevated plus maze. **(E)** *Fmr1*-KO mice (*n* = 10) were significantly impaired in the novel object recognition task (i.e., lower discrimination index) following a 1 h intertrial interval between sample and test phases when compared to WT controls (*n* = 9). **(F)** *Fmr1*-KO mice (*n* = 10) buried significantly more marbles across a 30-min marble burying test when compared to WT controls (*n* = 9). **(G)** In the social discrimination phase of the 3-chamber sociability test, WT mice (*n* = 10) showed a significant preference for a novel mouse over an empty cage. *Fmr1*-KO mice (*n* = 12) had no preference, exploring both test chambers for a similar amount of time. **(H)** Neither WT (*n* = 10) nor *Fmr1*-KO (*n* = 12) mice showed a significant preference for a novel or a familiar mouse in the social recognition phase of the 3-chamber sociability test. Data are expressed as mean ± SEM. Unpaired *T*-test, *p < 0.05; **p < 0.01; and ***p < 0.001 versus control conditions. See results for more details on the statistical results.

Once the new breeding colony was established at our animal facility, the behavioral phenotype was characterized to ensure that the expected traits were present (Figure 2). A battery of tests was performed to measure locomotor activity (open field test), anxiety-like behavior (open field test, elevated plus maze), learning and memory (novel object recognition task), perseverative behavior (marble burying task), and social behavior (3-chamber sociability test). The tests were performed across two cohorts to reduce the total number of behavioral tests performed by each mouse.

*Fmr1*-KO mice consistently showed significant hyperlocomotion in the open field test with this phenotype detectable in both cohorts (*t_(18)_* = 3.157; *p* < 0.01 and *t_(20)_* = 3.967; *p* < 0.001; Figures 2A,B, respectively). However, *Fmr1*-KO mice did not show any anxiety-like behavior based either on time spent in the center of the open field (*t_(18)_* = 9.975; *p* > 0.05; Figure 2C) or in time spent on the open arms of the elevated plus maze (*t_(18)_* = 1.518; *p* > 0.05; Figure 2D). *Fmr1*-KO mice had impaired recognition memory as measured in the novel object recognition task (*t_(17)_* = 2.324; *p* < 0.05 after a 1 h intertrial interval; Figure 2E). In our hands, *Fmr1*-KO mice showed a significant phenotype in two of the benchmark tasks used preclinically to compare to FXS in humans. Perseverative and stereotypic behavior was significantly higher in *Fmr1*-KO mice in the marble burying task (*t_(17)_* = 2.010; *p* < 0.05; Figure 2F). In the 3-chamber sociability test, a two-way ANOVA with cage condition (empty vs. containing a stranger mouse) and genotype (WT vs. Fmr1-KO) as factors revealed a significant main effect of cage condition (*F_(1,20)_* = 18.53, *p* < 0.001), but no significant main effect of genotype and no interaction effect. *Post hoc* analysis confirmed that *Fmr1*-KO mice were significantly impaired in the social discrimination phase, showing no preference for the novel stimulus mouse over the empty cage. In contrast, WT controls displayed the expected preference for the stimulus mouse (*p* < 0.001; Figure 2G). Neither genotype showed a preference in the social recognition trial (novel versus familiar stimulus mice; Figure 2H).

### 3.2 *Fmr1*-KO mice show impairments in microtubule dynamics

**Figure 3:**
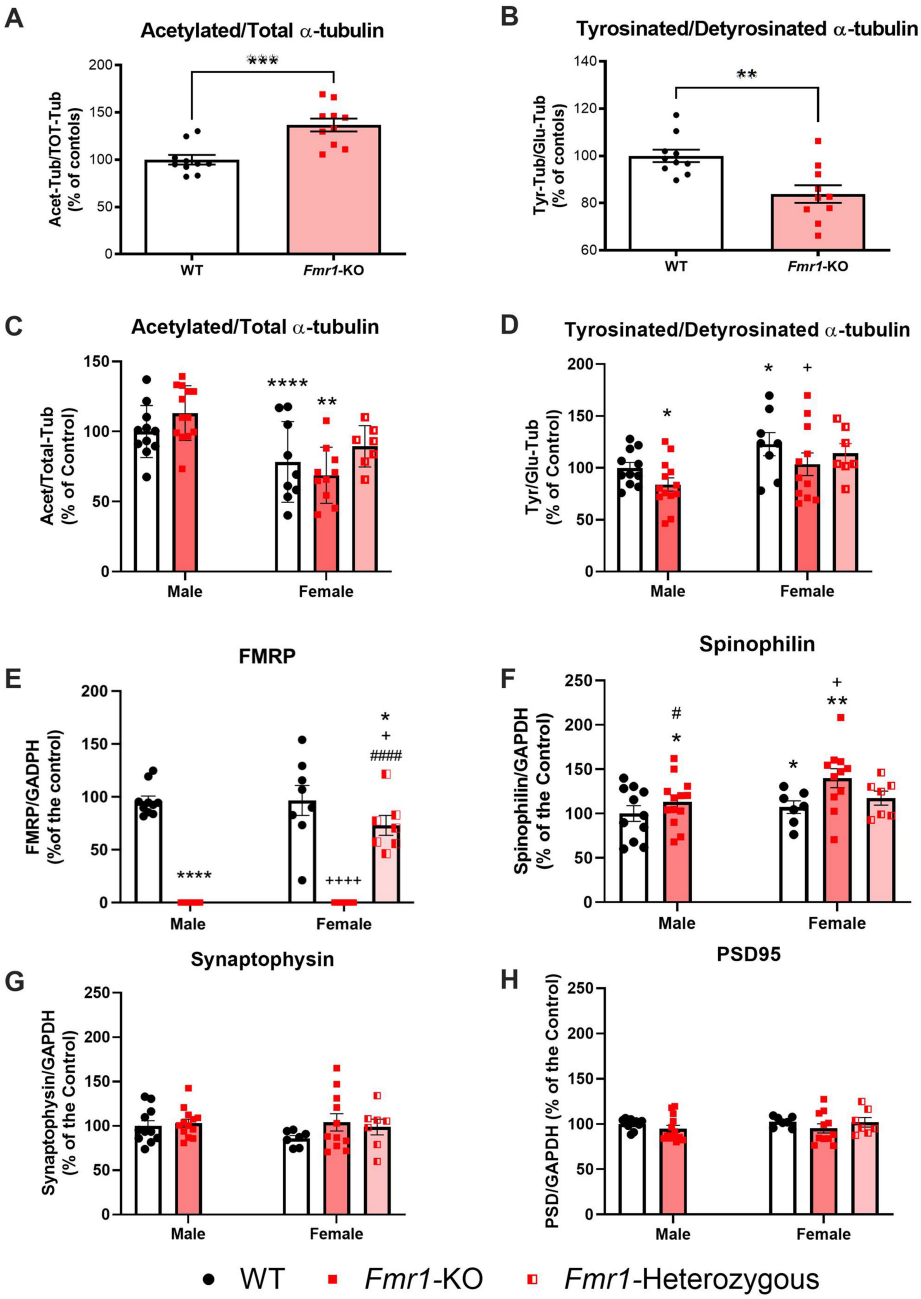
*Fmr1*-KO mice show significantly altered microtubule dynamics in the hippocampus. **(A)** male Fmr1-KO mice (*n* = 10) had significantly higher Acet/Tot-Tub *α*-tubulin ratios compared to WT (*n* = 10), and **(B)** significantly lower Tyr/Glu-Tub *α*-tubulin ratios. There were also sex-dependent effects for both Acet/Tot-Tub and Tyr/Glu-Tub *α*-tubulin. **(C)** Female WT (*n* = 9) and *Fmr1*-KO (*n* = 10) mice had significantly lower Acet/Tot-Tub compared to their respective genotype-matched males [WT (*n* = 11) and *Fmr1*-KO (*n* = 13) mice] while heterozygous females (*n* = 7) had similar Acet/Tot-Tub *α*-tubulin ratios to WT males. **(D)** Tyr/Glu-Tub levels are significantly higher in female WT (*n* = 8) and *Fmr1*-KO (*n* = 11) mice compared to their respective genotype-matched males [WT (*n* = 11) and *Fmr1*-KO (*n* = 13) mice], but *Fmr1*-KO mice of both sexes show significant reductions in Tyr/Glu ratios compared to WTs. Heterozygous females (*n* = 7) were not significantly different to WT males. **(E)** Complete KO of the *Fmr1* gene prevents FMRP expression as expected but heterozygous mice produce approximately 73% of WT FMRP levels [male: WT (*n* = 11) and *Fmr1*-KO (*n* = 13)] mice; [female: WT (*n* = 9), *Fmr1*-KO (*n* = 10) and *Fmr1*-heterozygous (*n* = 7)]. **(F–H)** Expression of synaptic markers [male: WT (*n* = 11) and *Fmr1*-KO (*n* = 13)] mice; [female: WT (*n* = 9), *Fmr1*-KO (*n* = 10) and *Fmr1*-heterozygous (*n* = 7)]. **(F)** The postsynaptic protein spinophilin is significantly increased in *Fmr1*-KO mice but produced at levels similar to WT by *Fmr1*-heterozygous females. There are no significant differences in **(G)** the presynaptic protein synaptophysin or **(H)** the postsynaptic protein PSD-95. Data are expressed as MEAN ± SEM. **(A,B)** Unpaired *T*-test, **p < 0.01; ***p < 0.001 versus WT controls. **(C–H)** Two-way ANOVA with sex (male, female) and genotype (WT, heterozygous, KO) as factors, *p < 0.05; **p < 0.01; ****p < 0.0001 versus male WT controls. ^+^*p* < 0.05; ^++++^*p* < 0.0001 versus WT females. ^#^*p* < 0.05; ^####^*p* < 0.0001 versus *Fmr1*-KO females.

Microtubule dynamic status in both *Fmr1*-KO and WT mice was compared using changes in the Acet/Tot-Tub and Tyr/Glu-Tub. In male mice, *Fmr1*-KO was associated with significant increase in Acet/Tot (*t_(18)_* = 4.310; *p* < 0.001; Figure 3A) and a significant decrease in the Tyr/Glu-Tub (*t_(18)_* = 3.513; *p* < 0.01; Figure 3B) compared to C57BL6/J controls, indicative of a shift toward a reduction in microtubule dynamics. Representative western blots are shown in the supplementary material as Supplementary Figures S1A,B. For the mixed-sex cohort, littermates comprised homozygous *Fmr1*-KO females, heterozygous females, *Fmr1*-KO males and WT males. A two-way ANOVA with sex (male, female) and genotype (WT, heterozygous, KO) as factors was conducted to assess the effects of sex and genotype on *α*-tubulin PTMs, FMRP, and synaptic markers. For the Acet/Tot-Tub, there was a significant effect for sex (*F_(1,46)_* = 26.51; *p* < 0.001) but there was no significant effect found for genotype (*F_(2,46)_* = 1.572; *p* > 0.05) – however, male mice did show the same tendency toward an increase in the Acet/Tot-Tub as seen in the previous cohort though it did not reach significance here (Figure 3C, Supplementary Figure S1C). *Post hoc* analyses revealed that both WT (*p* < 0.0001) and *Fmr1*-KO female mice (*p* < 0.01) had a significantly lower Acet/Tot-Tub compared to WT males, and *Fmr1*-KO females were also significantly lower than *Fmr1*-KO males (*p* < 0.001). However, heterozygous females were not significantly different to WT males, pointing to a complex relationship between genotype and sex on *α*-tubulin PTMs in these mice (Figure 3C; Supplementary Figure S1C). For Tyr/Glu *α*-tubulin, there were significant effects for both sex and genotype. WT females had significantly higher Tyr/Glu ratios compared to WT males, and both male and female *Fmr1*-KO mice show significantly lower levels of Tyr/Glu *α*-tubulin compared to WT males (Figure 3D; Supplementary Figure S1D). As with Acet/Tot-Tub, females heterozygous for *Fmr1* were not significantly different in Tyr/Glu *α*-tubulin compared to WT males (Figure 3D; Supplementary Figure S1D). In order to better understand the relationship between microtubules and other elements of brain biology, FMRP levels were measured which showed a significant effect for genotype (*F_(2,45)_* = 132.9; *p* < 0.0001) but no significant effect for sex. As expected, full *Fmr1*-KO animals of both sexes produces no detectable FMRP (Figure 3E; Supplementary Figure S1E). Heterozygote females expressed levels of FMRP that were approximately 73% of those seen in WT animals (Figure 3E; Supplementary Figure S1E) – this value was significantly lower than male and female WT mice (*p* < 0.05 in both cases) and significantly higher than *Fmr1*-KO females (*p* < 0.0001). Finally, a panel of pre- (synaptophysin) and post-synaptic (PSD-95 and spinophilin) molecular markers were measured to determine whether disrupted microtubule dynamics in this mouse model were associated with altered synaptic plasticity (Supplementary Figure S1E). Out of the three markers analyzed, only the postsynaptic protein spinophilin was significantly affected by *Fmr1* mutation (Figure 3F). There were significant effects both for sex (*F_(1,45)_* = 4.469; *p* < 0.05) and genotype (*F_(2,45)_* = 3.210; *p* < 0.05). *Fmr1*-KO mice expressed significantly higher levels of spinophilin compared to their WT controls (*p* < 0.05 for both males and females), but heterozygous females expressed similar levels to WT females controls – indicating that there were no detectable differences in spinophilin once one functional copy of *Fmr1* is present. Interestingly, there was a slight but significant difference between WT males and WT females, with females expressing significantly higher levels of spinophilin compared to males (*p* < 0.05). There were no effects for sex or genotype in either synaptophysin (Figure 3G) or PSD-95 (Figure 3H).

### 3.3 GSK-3β inhibition affects behavior in *Fmr1*-KO mice

**Figure 4:**
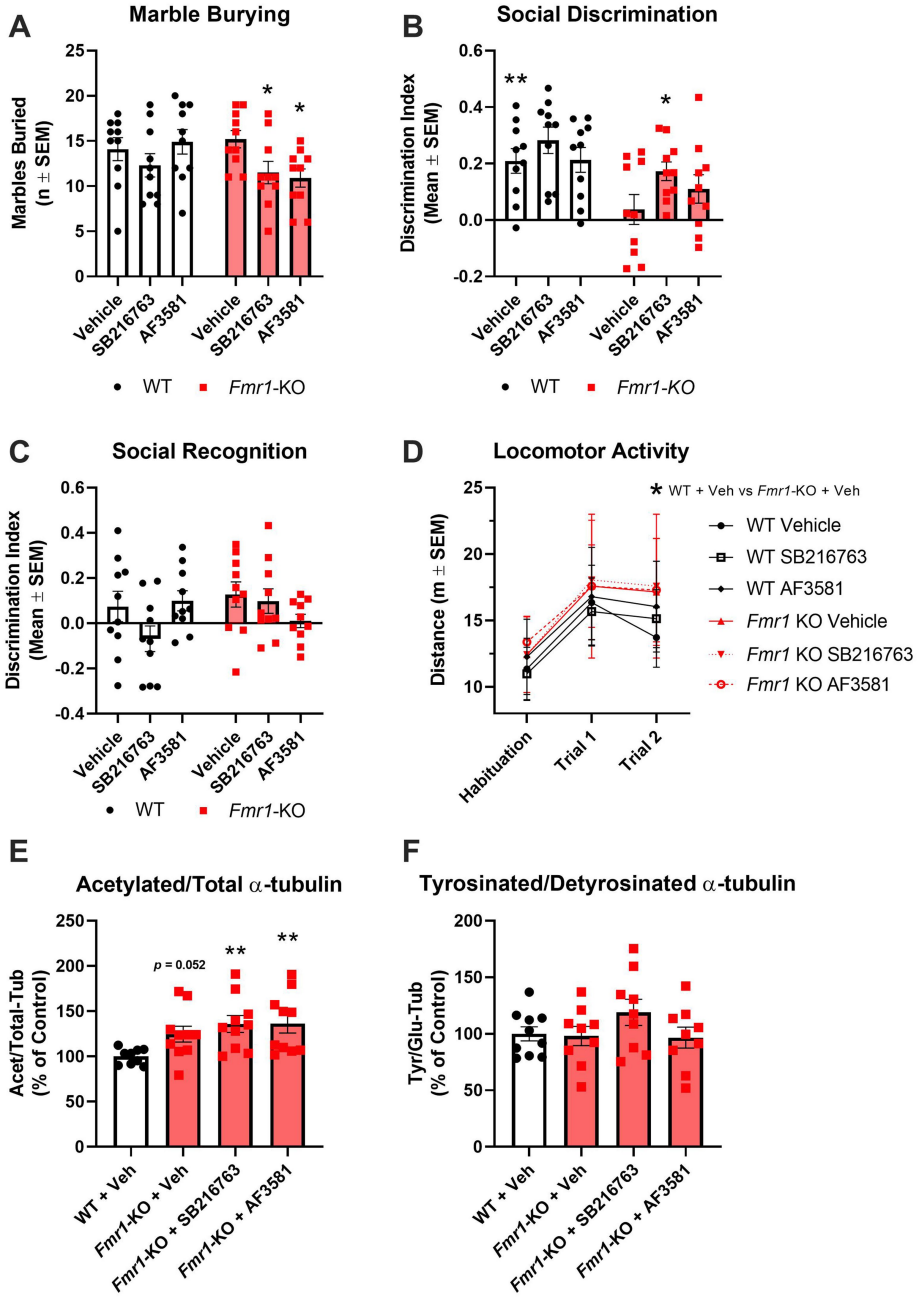
Effects of GSK-3β inhibition on behavioral and molecular endpoints in *Fmr1*-KO mice. **(A–D)** Behavioral readouts [WT: Vehicle (*n* = 10), SB216763(*n* = 10) and AF3581 (*n* = 10); *Fmr1*-KO: Vehicle (*n* = 10), SB216763 (*n* = 10) and AF3581 (*n* = 10)]. **(A)** Perseverative behavior was rescued by GSK-3β inhibition as marble burying was significantly reduced in *Fmr1*-KO mice following treatment with SB216763 and AF3581. **(B)** Vehicle-treated *Fmr1*-KO mice were significantly impaired in the social discrimination trial of the 3-chamber sociability test compared to WT, this was significantly rescued by SB216763 and partially by AF3581. **(C)** Social recognition was not different in *Fmr1*-KO mice nor did GSK-3β inhibition have any effect on this behavior. **(D)** Overall, *Fmr1*-KO mice showed significantly higher levels of locomotor activity compared to WT mice. Only vehicle-treated *Fmr1*-KO mice showed a specific hyperlocomotion, *Fmr1*-KO mice treated with either SB216763 or AF3581 were not significantly different to WT mice at any time point. **(E)** All three *Fmr1*-KO treatment groups had higher Acet/Tot-Tub compared to vehicle-treated WT mice, with both SB216763- and AF3581-treated *Fmr1*-KO mice showing significantly higher expression. **(F)** There were no significant differences between Tyr/Glu *α*-tubulin ratios in any of the treatment groups, though SB216763-treated *Fmr1*-KO mice were trending toward an increase compared to WT and *Fmr1*-KO vehicle controls. Data are expressed as MEAN ± SEM. **(A-C)** Two-way ANOVA with genotype (WT, *Fmr1*-KO) and treatment (vehicle, SB216763, AF3581) as factors, *p < 0.05; **p < 0.01 versus vehicle-treated *Fmr1*-KO mice. **(D)** Three-way repeated measures ANOVA with genotype (WT, *Fmr1*-KO) and treatment (vehicle, SB216763, AF3581) as between-subjects factors, and trial (habituation, Trial 1 and Trial 2) as a within-subjects factor, *p < 0.05; **p < 0.01 versus vehicle-treated *Fmr1*-KO mice. **(E,F)** One-way ANOVA with treatment groups (vehicle, SB216763, AF3581) as the between-subjects factor, **p < 0.01 versus vehicle-treated WT mice.

All mice in the GSK-3β inhibitors arm of the study were males and treated once a day for 10 days with SB216763 or AF3581. Behavioral (marble burying test and 3-Chamber Sociability test) and hippocampal molecular (Acet/Tot-Tub and Tyr/Glu *α*-tubulin ratios) analysis were performed. A two-way ANOVA with genotype (WT, *Fmr1*-KO) and treatment (vehicle, SB216763, AF3581) as factors was conducted to assess the effect of treatments on behavioral readouts. Marble burying was performed 9 days after treatment. There was a significant effect of treatment on marble burying (*F_(1,36)_* = 5.245; *p* = 0.0280). However, there was no significant effect of genotype nor an interaction effect found in this experiment. *Post hoc* analysis highlighted that *Fmr1*-KO mice treated with SB216763 or AF3581 buried significantly (*p* < 0.05) fewer marble during the test compared to their vehicle-treated *Fmr1*-KO counterparts (Figure 4A). There were no significant differences in the WT groups. At day 10 of treatment, *Fmr1*-KO mice were tested in the 3-Chamber Sociability Test. Following a 5-min habituation trial, all mice underwent two 10-min test trials. There were clear differences due to genotype and treatment on the sociability scores obtained in the 3-Chamber Sociability Test. Two-way ANOVA confirmed a significant effect for genotype on discrimination index (*F_(2,54)_* = 11.91; *p* = 0.0011) but no effect of treatment nor an interaction effect was found. *Post hoc* analysis confirmed that vehicle-treated *Fmr1*-KO mice showed a significantly (*p* < 0.01) lower preference compared to WT mice (Figure 4B). Treating *Fmr1*-KO mice with SB216763 significantly increased their discrimination index scores compared to vehicle-treated *Fmr1*-KO mice (*p* < 0.05), changes in AF3581-treated *Fmr1*-KO mice were similar but lacked to reach significance (Figure 4C). In Trial 2 (Social Recognition), neither WT nor *Fmr1*-KO mice showed a clear preference for the novel over the familiar mouse and treatments had no effects in either genotype (Figure 4D). Locomotor activity was measured across the habituation period and the two trials of the sociability test. A three-way repeated measures ANOVA was conducted with genotype (WT, *Fmr1*-KO) and treatment (vehicle, SB216763, AF3581) as between-subjects factors, and trial (habituation, Trial 1 and Trial 2) as a within-subjects factor. Significant main effects of genotype (*F_(1,54)_* = 5.30, *p* = 0.0252) and trial (*F_(2,108)_* = 78.43, *p* < 0.0001) were observed, with *Fmr1*-KO mice showing overall increased locomotor activity. No significant main effect of treatment or interaction effects between factors were found. Post hoc analysis revealed a significant difference between vehicle-treated *Fmr1*-KO and WT mice only during Trial 2 (Figure 4D).

The impact of GSK-3β inhibition on hippocampal dynamics was assessed using one-way ANOVA with treatment groups (vehicle, SB216763, AF3581) as the between-subjects factor. For the Acet/Total *α*-tubulin ratio, a main effect of treatment was observed (*F_(3,35)_* = 3,751, *p* = 0.0195), with KO vehicle-treated mice showing a trend toward higher values compared to WT vehicle-treated mice (*p* = 0.052; Figure 4E; Supplementary Figure S1G). Neither SB216763 nor AF3581 had any effect on Acet/Tot-Tub in *Fmr1*-KO mice which were both significantly higher than WT vehicle-treated mice (*p* < 0.01 in both cases; Figure 4E; Supplementary Figure S1G). For the Tyr/Glu *α*-tubulin ratio, no significant group differences were found (*F_(3,33)_* = 1.332, *p* = 0.2805), although a non-significant trend was noted following SB216763 treatment (Figure 3F; Supplementary Figure S1H).

## 4 Discussion

### 4.1 Rationale for experimental design and tissue selection

We set out to bridge behavior, cytoskeletal biochemistry and pharmacology in the *Fmr1*-KO mouse, asking whether inhibition of GSK-3β could improve key behavioral abnormalities and normalize *α*-tubulin PTMs. In this study, we focused our molecular analyses on the hippocampus, given its well-established involvement in cognitive and social processes assessed through the behavioral tasks employed, such as novel object recognition and social discrimination. While other tasks, including marble burying and elevated plus maze, recruit broader cortico-limbic circuits, the hippocampus plays a modulatory role in anxiety-related and exploratory behaviors (Zemla and Basu, 2017). The decision to conduct behavioral testing exclusively in male mice, while including both sexes in the molecular analyses, was based on scientific, ethical, and logistical considerations. Behavioral studies in females are more variable due to the influence of the oestrous cycle, and reliable tracking would have significantly increased the number of animals required. In contrast, molecular analyses allowed us to explore sex differences more directly, using a mixed-sex cohort derived from the same litters. This approach enabled the detection of genotype- and sex-dependent effects while minimizing animal use, in accordance with the principles of the 3Rs.

### 4.2 Summary of main findings

In this study, we found that adult male *Fmr1*-KO mice displayed behavioral deficits in object recognition, social discrimination, and stereotypic/perseverative traits. At the molecular level, these animals showed altered *α*-tubulin PTMs in the hippocampus, with increased acetylation and reduced tyrosinated/detyrosinated tubulin ratios. Mixed-sex molecular analyses further revealed sex- and genotype-dependent differences in tubulin *α*-tubulin PTMs and synaptic markers, including elevated spinophilin levels in *Fmr1*-KO females. Finally, GSK-3β inhibition with chronic administration of SB216763 and AF3581 led to partial behavioral rescue, but did not reverse the molecular abnormalities in *α*-tubulin PTMs.

### 4.3 Behavioral phenotypes and hippocampal *α*-tubulin PTMs profile in male *Fmr1*-KO mice

In our study, *Fmr1*-KO mice (aged 3 months) showed hyperactivity in the open field, recognition memory impairments in the NOR, increased stereotypic/perseverative behavior and social discrimination deficits. These are all behavioral traits consistent with those seen in individuals with FXS and previously reported in the *Fmr1*-KO literature despite some important variability (Kat et al., 2022). However, these behavioral traits do not fully reproduce FXS symptomology as we failed to find evidence of an anxiety phenotype since *Fmr1*-KO mice not showing any tendency to avoid the center area of the open field or the open arms of the elevated plus maze. Anxiety is a core feature of FXS and other authors have reported reduced anxiety in the elevated plus maze previously (Eadie et al., 2009). This finding confirms again that variability in behavioral results between laboratories using the *Fmr1*-KO mouse model has been a major limitation in developing treatments for FXS (Kat et al., 2022). Untreated males *Fmr1*-KO that underwent behavioral phenotyping showed the canonical triad of hyperactivity, perseveration and impaired recognition memory together with an hippocampal signature that typifies reduced microtubule dynamics, an increase in Acet/Total-Tub and a decrease in the Tyr/Glu-Tub ratio. When we generated a mixed-sex cohort for molecular analysis by crossing *Fmr1*-KO males with heterozygous females we found a similar *α*-tubulin PTMs signature in *Fmr1*-KO male, compared to male WT, with a confirmed statistically decreased Tyr/Glu-Tub ratio. However, Acet/Total-Tub only showed a tendency toward increased levels that lacked to reach statistical significance. This apparent discrepancy with the results obtained in the first cohort of animals may be explained with the additional handling and stimuli that the first cohort of mice had during the different behavioral tests. Thus, we have previously shown that handling can affect expression of *α*-tubulin PTMs in the rat hippocampus (Bianchi et al., 2009).

### 4.4 Sex differences in hippocampal *α*-tubulin PTMs profile, *Fmr1* and synaptic marker expression

Interestingly, hippocampal Acet/Total-Tub expression and Tyr/Glu-Tub ratio varied with sex in both WT and *Fmr1*-mutant mice. Thus, female WT mice showed lower Acet/Total-Tub and higher Tyr/Glu-Tub ratio compared to male WT indicating increased microtubule dynamics. This observation of sex-related differences in the expression of *α*-tubulin PTMs at baseline is corroborated by our previous clinical data showing that circulating levels of acetylated *α*-tubulin are lower in female healthy volunteers compared to males (Colic et al., 2019). The reasons for such differences are not yet elucidated, but they may relate to the distinction in circulating levels of steroidal hormones and in particular of neurosteroids. Hence, neurosteroids have been shown to modulate neuronal microtubule dynamics and neuronal plasticity (Bianchi and Baulieu, 2012; Raciti et al., 2023). Moreover, HDAC6, the principal cytoplasmic tubulin de-acetylase, is encoded on the X chromosome (mouse X A3; human Xp11.23) and partially escapes X-inactivation. Consequently, females may be predicted to express a modestly higher effective dose of HDAC6, which might lower basal *α*-tubulin acetylation. Noteworthy, data showing important sex-dependent differences in dendritic and synaptic plasticity phenomena at different anomical and cellular levels have been reported (Hyer et al., 2018). Specific studies, will be required to further elucidate the underlying mechanisms of this interesting gender difference in central and peripheral *α*-tubulin PTMs expression. Some sex-dependent differences in the expression of hippocampal *α*-tubulin PTMs were also shown in Fmr1-mutants with Acet/Total-Tub appearing to have the tendency do decrease in *Fmr1*-KO female compared to WT female, while the opposite was observed in male mice. However, the observed decrease in Acet/Total-Tub in *Fmr1*-KO female lacked to reach statistical significance and it has to be interpreted with caution. Levels of Tyr/Glu-Tub ratio were statistically decreased in *Fmr1*-KO female compared to WT female, which is consistent with what was observed in *Fmr1*-KO male when compared to WT male. This finding suggests that loss of FMRP shifts microtubules toward a less dynamic state, dysregulating synaptic plasticity and spine remodeling. The observed increase in spinophilin expression observed in both male and female *Fmr1*-KO is in line with the morphological changes in dendritic spines observed in the mature synapses of this mice (Comery et al., 1997; Nimchinsky et al., 2001). Noteworthy, similar morphological changes were consistently observed in post-mortem analysis of human FXS brain tissue (Rudelli et al., 1985; Wisniewski et al., 1991; Irwin et al., 2000).

### 4.5 Genotype-by-sex interactions and potential microtubule regulatory mechanisms

The potential inversion of the Acet/Total-Tub signal in *Fmr1*-KO females adds a further layer of complexity. As stated above, the mechanisms behind sex-dependent differences in *α*-tubulin PTMs are not clear yet, but it is possible to speculate an hypothetical explanations for what it has been observed in *Fmr1*-mutants. αTAT1 catalyzes lys-40 acetylation on polymerized *α*-tubulin and is the enzymatic counterpart of HDAC6. Ribosome-profiling and HITS-CLIP data show that *αTat1* mRNA is a direct, high-affinity FMRP target and is over-translated in male *Fmr1*-KO cortex (Darnell et al., 2011), predicting higher αTAT1 protein when FMRP is absent. However, direct αTAT1 protein measurements in hippocampus *Fmr1*-KO have not been performed yet. However, it is plausible that in *Fmr1*-KO males the αTAT1 increase dominates and Acet/Total-Tub rises, whereas in homozygous *Fmr1*-KO females the relative excess of HDAC6 counter-balances αTAT1, yielding the lower Acet/Total-Tub baseline and the apparent inversion. On the other hand, *α*-tubulin detyrosination is governed by the autosomal TTL/vasohibin-SVBP cycle (Aillaud et al., 2017) and is therefore unaffected by sex-chromosome dosage which might explaining why Tyr/Glu-Tub declines to a similar extent in both sexes. It has to be noted that these intertwined enzyme and dosage effects, layered on the variable 129 flanking DNA present in our B6.129P2 line, may also account for cohort-to-cohort fluctuations in Acet/Total-Tub and reinforce the need to control both genetic background and sex when *α*-tubulin PTMs are used as biomarkers. Both WT and heterozygous *Fmr1*-KO females mice displayed higher spinophilin immunoreactivity than *Fmr1*-KO males. Spinophilin is highly enriched in dendritic spines, and its abundance scales positively with spine number during development (Feng et al., 2000). Although a direct causal relationship between tubulin acetylation and spinophilin has not been demonstrated *in vivo*, knock-down of αTAT1 in cultured hippocampal neurons reduces tubulin acetylation (Kalebic et al., 2013). Together these findings support a speculative model in which diminished *α*-tubulin acetylation biases spine remodeling toward an immature, spinophilin-rich state, providing a plausible explanation for the elevated spinophilin observed in female mutants. Finally, the current study shows that the presence of a single copy of *Fmr1* in heterozygous female mice is e protective, despite a 27% decrease in FMRP levels. Thus, *Fmr1*-heterozygous female mice show readings on all molecular measures comparable to WT female mice.

### 4.6 Effects of GSK-3β inhibition on behavioral endpoints and hippocampal *α*-tubulin PTMs profile

Increasing evidence indicate microtubule dynamics and acetylated *α*-tubulin as potential pharmacological target in neurodevelopmental disorders (Bonini et al., 2017; Graziella and Daniele, 2020). This concept is supported by findings in another X-linked neurodevelopmental disorder, CDKL5 deficiency disorder (CDD), where microtubules have also been implicated as a driver of pathology (Barbiero et al., 2022). Treatment of *Cdkl5*-KO mice with pregnenolone methyl ether (PME), a direct microtubule modulator, rescued both synaptic and behavioral deficits (Barbiero et al., 2022). FMRP and GSK-3β interact with microtubules via the microtubule-associated protein 1B (MAP1B) (Lu et al., 2004; Barnat et al., 2016; Antar et al., 2005). Neurite growth in neurons requires inactivation of GSK-3β to promote microtubule organization (Zhou et al., 2004). In *Fmr1*-KO mice, microtubules tend toward a less dynamic state and are resistant to nocodazole-induced shifts toward a more dynamic state (Lu et al., 2004). This opens to the possibility of using strategies to target microtubules in FXS in order to promote a more dynamic microtubule state and thus promote synaptic plasticity.

Here, we explored whether a 10-day systemic regimen of the GSK-3β inhibitors SB216763 or AF3581 improves abnormal behaviors and shifts *α*-tubulin PTMs in male *Fmr1*-KO mice. SB216763 and AF3581 are here tested for the first time in *Fmr1*-KO mice performing the Marble Burying Test or the 3-Chamber Sociability Test. SB216763 has been shown to have a benefit on audiogenic seizures and hyperlocomotion in the *Fmr1*-KO mouse model (Min et al., 2009; Westmark et al., 2021; Guo et al., 2012). In the Marble Burying Test, *Fmr1*-KO mice typically bury more marbles than their WT controls (Veeraragavan et al., 2012; Schaefer et al., 2021; Bausch et al., 2018). We confirmed this increased perseverative behavior in our first cohort of untreated male *Fmr1*-KO. However, male *Fmr1*-KO receiving daily vehicle injections did not differ from WT mice subjected to the same regimen, mirroring other reports in which vehicle administration abolished the marble burying phenotype (Veeraragavan et al., 2011; Thomas et al., 2012). Despite this vehicle effect, both GSK-3*β* inhibitors significantly reduced marble burying in *Fmr1*-KO, indicating that GSK-3β inhibition can modulate perseverative behavior in this model. In our study, neither SB216763 nor AF3581 affected marble burying in WT mice, in contrast to agents such as JNJ16259685 (an mGluR1 antagonist) and dicyclomine (an M₁/M₃ muscarinic antagonist), which decrease marble burying in both WT and KO animals (Veeraragavan et al., 2011; Thomas et al., 2012). These results underscore the specificity of GSK-3β as a therapeutic target for FXS. In the 3-Chamber Sociability Test, *Fmr1*-KO mice consistently exhibit impaired social discrimination (Bausch et al., 2018; Hou et al., 2021; Rosenheck et al., 2021). We confirmed this deficit in both our untreated and vehicle-treated *Fmr1*-KO cohorts: these mice showed no preference for the stranger mouse over the empty cage. GSK-3β inhibition rescued this impairment, with SB216763 apparently demonstrating better efficacy than AF3581, since the latter failed to reach statistical significance. Porceddu et al. (2021) previously reported that AF3581 at the same dose used here (10 mg/kg, i.p.), but administered twice daily for 21 days rather than once daily for 10 days, prevented hyperactivity, sensorimotor deficits and social avoidance in *Fmr1*-KO mice. It is therefore plausible that our shorter, once-daily regimen underlies the partial recovery we observed with AF3581. Importantly, neither compound affected sociability in WT mice. Nevertheless, lithium also significantly improves 3-chamber performance in *Fmr1*-KO mice Supporting the utility of GSK-3 inhibition in FXS (Mines et al., 2010).

In the pharmacological experiment, expression of hippocampal Acet/Total-Tub was still increased in *Fmr1*-KO mice with a trend very close to significance when compared to WT counterparts, while Tyr/Glu-Tub was not found decreased as it was observed in the first cohort of untreated animals. This discrepancy can be ascribed to variability in the cohort, but it should be noted that we have previously shown that repeated handling procedures and daily injections of vehicle flattened the hippocampal *α*-tubulin PTM phenotype in rats isolated from the time of weaning (Bianchi et al., 2009). Our recent observation indicates that other rodent brain regions like the mPFC and amygdala might be less sensitive to handling and injection procedures in models of disease (data not published). GSK-3β inhibitors did not show any effect on Acet/Total-Tub. Thus, treated *Fmr1*-KO still have significant increased levels of this *α*-tubulin PTMs compared to WT. Tyr/Glu-Tub was also not significantly affected by treatments. However, a trend toward higher Tyr/Glu-Tub after SB216763 are in line with *in vitro* data linking GSK-3 inhibition to enhanced tubulin detyrosination (Barnat et al., 2016). It might be possible that, if an effect on Tyr/Glu-Tub was present, it was masked by the fact the *Fmr1*-KO did not present the decreased Tyr/Glu-Tub phenotype showed by the untreated cohort. Our current data indicate that GSK-3β inhibitors do not affect *α*-tubulin PTMs in the hippocampus of *Fmr1*-KO. The results are surprising considering the well documented, and previously discussed here, modulatory activity of the GSK-3β pathway on microtubule dynamics.

## 5 Conclusion

In conclusion, the current findings demonstrate that GSK-3β inhibition can rescue key behavioral deficits in *Fmr1*-KO mice, including perseveration and impaired sociability, supporting its potential as a therapeutic target in FXS. Although *α*-tubulin PTMs, such as Acet/Total-Tub and Tyr/Glu-Tub, were altered in untreated *Fmr1*-KO mice, GSK-3β inhibitors did not restore these molecular markers in the hippocampus.

### 5.1 Limitations and future directions

This study was conducted during the COVID-19 pandemic, under restricted access to breeding colonies and laboratory space. These constraints, along with the absence of additional dedicated funding, limited our ability to implement a fully powered, sex-balanced design across all experimental arms.

This study presents a multi-layered characterization of behavioral, molecular, and pharmacological phenotypes in the *Fmr1*-KO mouse model of FXS, but several limitations and future directions should be acknowledged.

First, the pharmacological experiments were conducted exclusively in male mice, limiting the generalisability of the findings across sexes. Our molecular analyses clearly revealed sex-dependent differences in *α*-tubulin PTMs. These findings highlight the importance of considering sex as a biological variable, and suggest that sex-specific responses to GSK-3β inhibition cannot be excluded. Future studies should explicitly investigate whether female Fmr1-KO mice respond differently to pharmacological intervention and whether sex-stratified treatment approaches may be warranted.

Second, although both SB216763 and AF3581 demonstrated behavioral efficacy in male *Fmr1*-KO mice, rescuing perseveration and sociability deficits to varying degrees, the study was not designed to directly compare the two compounds in a head-to-head fashion in several behavioral and molecular endpoints. Further comparative studies are required to define the relative efficacy, dosing regimens, and molecular targets of SB216763 and AF3581 in the FXS context.

Additionally, the molecular arm of the pharmacological study focused exclusively on the hippocampus. While this choice was aligned with the behavioral tasks employed, recent observations suggest that other brain regions, such as the medial prefrontal cortex or amygdala, may be less sensitive to repeated handling and injection procedures that can mask molecular phenotypes in the hippocampus. This regional specificity warrants further investigation to better localize drug effects on microtubule dynamics and downstream synaptic changes.

Finally, although *α*-tubulin PTMs are emerging as promising biomarkers for neurodevelopmental disorders, their interpretation requires strict control over factors such as genetic background, sex, environmental stressors, and tissue region. Our results reinforce the need for rigorous experimental design and stratification when assessing cytoskeletal biomarkers in translational studies.

Taken together, this work lays important groundwork for future research aimed at integrating behavioral, molecular, and sex-specific data to inform personalized therapeutic approaches in FXS and related conditions.

## Data Availability

The raw data supporting the conclusions of this article will be made available by the authors, without undue reservation.

## Supplementary material

SUPPLEMENTARY FIGURE S1 Representative multiplexed infrared Western Blots of hippocampal lysates. **(A,B)** Untreated male WT and *Fmr1*-KO mice; **(A)** Detection of acetylated and total α-tubulin; **(B)** Detection of tyrosinated and detyrosinated α-tubulin. **(C-F)** Untreated WT male, *Fmr1*-KO male, WT female, *Fmr1*-KO female and *Fmr1*-heterozygous female mice; **(C)** Detection of acetylated and total α-tubulin; **(D)** Detection of tyrosinated and detyrosinated α-tubulin; **(E)** Detection of FMRP and GAPDH; **(F)** Detection of spinophilin, synaptophysin, PSD-95 and GAPDH. **(G,H)** male WT and *Fmr1*-KO mice treated with vehicle and GSK-3b inhibitors (SB216763 and AF3581); **(G)** Detection of acetylated and total α-tubulin; **(H)** Detection of tyrosinated and detyrosinated α-tubulin.
