# Supplementary figures and images for: Behavioral benefits of GSK-3β inhibition and state-dependent microtubule signatures in the Fmr1-KO mouse
Source: Front Neurosci. 2025 Oct 2;19:1643439. doi: 10.3389/fnins.2025.1643439 (PMC12528152; doi:10.3389/fnins.2025.1643439)

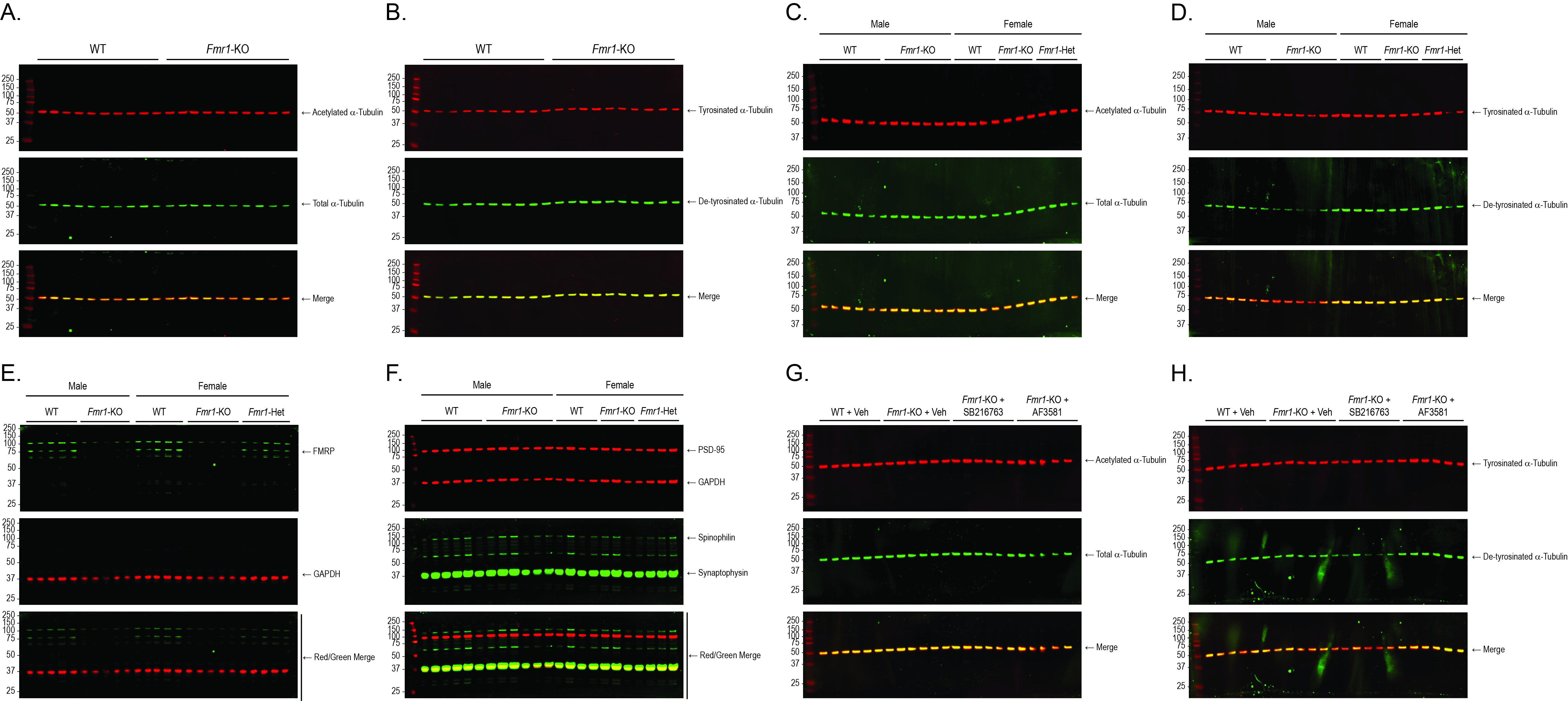

Supplement: SUPPLEMENTARY FIGURE S1 — Representative multiplexed infrared Western Blots of hippocampal lysates. (A,B) Untreated male WT and Fmr1-KO mice; (A) Detection of acetylated and total α-tubulin; (B) Detection of tyrosinated and detyrosinated α-tubulin. (C-F) Untreated WT male, Fmr1-KO male, WT female, Fmr1-KO female and Fmr1-heterozygous female mice; (C) Detection of acetylated and total α-tubulin; (D) Detection of tyrosinated and detyrosinated α-tubulin; (E) Detection of FMRP and GAPDH; (F) Detection of spinophilin, synaptophysin, PSD-95 and GAPDH. (G,H) male WT and Fmr1-KO mice treated with vehicle and GSK-3b inhibitors (SB216763 and AF3581); (G) Detection of acetylated and total α-tubulin; (H) Detection of tyrosinated and detyrosinated α-tubulin. [file Image_1.JPEG]
